# An Economic Evaluation of Home Management of Malaria in Uganda: An Interactive Markov Model

**DOI:** 10.1371/journal.pone.0012439

**Published:** 2010-08-27

**Authors:** Yoel Lubell, Anne J. Mills, Christopher J. M. Whitty, Sarah G. Staedke

**Affiliations:** 1 Mahidol-Oxford Tropical Medicine Research Unit, Mahidol University, Bangkok, Thailand; 2 Department of Public Health and Policy, London School of Hygiene and Tropical Medicine, London, United Kingdom; 3 Department of Infectious and Tropical Diseases, London School of Hygiene and Tropical Medicine, London, United Kingdom; Direccion General de Epidemiologia, Peru

## Abstract

**Background:**

Home management of malaria (HMM), promoting presumptive treatment of febrile children in the community, is advocated to improve prompt appropriate treatment of malaria in Africa. The cost-effectiveness of HMM is likely to vary widely in different settings and with the antimalarial drugs used. However, no data on the cost-effectiveness of HMM programmes are available.

**Methods/Principal Findings:**

A Markov model was constructed to estimate the cost-effectiveness of HMM as compared to conventional care for febrile illnesses in children without HMM. The model was populated with data from Uganda, but is designed to be interactive, allowing the user to adjust certain parameters, including the antimalarials distributed. The model calculates the cost per disability adjusted life year averted and presents the incremental cost-effectiveness ratio compared to a threshold value. Model output is stratified by level of malaria transmission and the probability that a child would receive appropriate care from a health facility, to indicate the circumstances in which HMM is likely to be cost-effective. The model output suggests that the cost-effectiveness of HMM varies with malaria transmission, the probability of appropriate care, and the drug distributed. Where transmission is high and the probability of appropriate care is limited, HMM is likely to be cost-effective from a provider perspective. Even with the most effective antimalarials, HMM remains an attractive intervention only in areas of high malaria transmission and in medium transmission areas with a lower probability of appropriate care. HMM is generally not cost-effective in low transmission areas, regardless of which antimalarial is distributed. Considering the analysis from the societal perspective decreases the attractiveness of HMM.

**Conclusion:**

Syndromic HMM for children with fever may be a useful strategy for higher transmission settings with limited health care and diagnosis, but is not appropriate for all settings. HMM may need to be tailored to specific settings, accounting for local malaria transmission intensity and availability of health services.

## Introduction

Prompt treatment with effective antimalarial drugs is one of the key strategies for reducing the burden of malaria. However, health-care infrastructure is often inadequate in Africa, limiting availability of diagnostics and malaria treatment [1], [2]. The World Health Organization (WHO) has promoted home management of malaria (HMM) as a major strategy to improve access to antimalarials [3], and eighteen African countries have adopted this policy [4]. HMM involves presumptively treating febrile children at or near home with pre-packaged antimalarials distributed by trained members of the community. The application of HMM strategies varies somewhat in different settings. In Uganda, volunteers from the community are trained to evaluate and treat febrile children and are provided with antimalarial drugs from the Ministry of Health to distribute free-of-charge. The community drug distributors presumptively provide antimalarials for treatment of febrile illnesses in young children, without confirmation using a diagnostic test. Although HMM aims to minimize barriers to care, there are potential downsides to this strategy [5]. Presumptive treatment of all febrile illnesses as malaria could result in poor health outcomes due to delays in treating non-malarial illnesses [6], unnecessary exposure to antimalarial medications and their toxicities [7], increased drug pressure and potential for parasite resistance [8], and wastage of valuable drugs reducing their cost-effectiveness [9]. In addition, HMM is a major and costly undertaking, requiring considerable investment [10], which may divert resources from other public health activities.

Despite widespread advocacy for HMM, data supporting the strategy are limited. The effectiveness and cost-effectiveness of HMM have not been fully established in many settings. The few available studies indicate that effectiveness varies depending on epidemiology, healthcare setting and drug resistance patterns, and very few studies have evaluated use of artemisinin-based combination therapies (ACTs) in HMM programmes [11]. Whether ACTs, which have been adopted as first-line treatment for uncomplicated malaria in most African countries, can be successfully incorporated into HMM and used safely and effectively is a critical question [5], [12].Two recent studies suggest that introducing ACTs into HMM programmes is feasible and acceptable, resulting in high utilization and increasing prompt appropriate treatment [4], [13]. However, currently there are no published data on the cost-effectiveness of ACTs in HMM programmes.

Uganda was the first country to adopt HMM launching the national home-based management of fever programme in 2002 [14]. Uganda's HMM programme appears to be welcomed by the community, and has been shown to increase the proportion of febrile children who receive prompt antimalarial treatment [14], [15], [16], [17], [18]. The Ugandan Ministry of Health plans to incorporate artemether-lumefantrine (AL), an ACT, into the HMM program, but distribution of this regimen has been limited by severe shortages of the drugs [19]. Deployment of ACTs within the public health sector already poses significant challenges to many countries [20], [21], and the costs and benefits of deploying of ACTs into HMM will need to be assessed.

HMM is likely to be most effective and cost-effective in areas with high malaria transmission, limited health care infrastructure, and poor access to antimalarial treatment. In a recent study conducted in Kampala, the urban capital of Uganda, for instance, provision of AL at home significantly improved the proportion of febrile children receiving prompt effective antimalarial treatment compared to conventional care, but only produced modest health benefits at the cost of substantial over-treatment [22]. These results suggest that HMM may not be appropriate for areas with lower malaria transmission and better access to care. These findings, however, are by no means generalizable to areas with different malaria transmission intensity, or where access to health care facilities might be more limited. Obtaining primary data from a broad range of settings on the other hand is logistically impractical. There is a need, therefore, for analytical tools to establish where HMM is expected to be cost-effective. To investigate further the cost-effectiveness of HMM in different settings, a Markov model was developed as a decision support tool to compare the cost-effectiveness of HMM to conventional care for febrile illnesses in children under five. The model allows the user to adjust certain input parameters, and produces output stratified by level of malaria transmission and the probability that a child would receive appropriate care from a health facility, indicating the circumstances in which HMM is likely to be cost-effective (Model S1).

## Methods

### Overview

The model is designed to compare the costs and health outcomes for children under five with febrile illness who benefit from an HMM programme to current conventional care for children without HMM. This comparison is made across different probability strata that a child will receive appropriate treatment for malaria and bacterial illnesses from existing health services, including correct diagnosis and effective treatment. This stratification aims to capture the differences between, for instance, urban areas where good quality health care might be available, and more remote areas where access to health care is limited or in extreme cases non-existent. For children in the HMM arm, all non-severe febrile illnesses are treated presumptively with an antimalarial regimen. If the antimalarial is ineffective in treating a true case of malaria, or if the cause of illness is other than malaria, the illness can become severe. In the conventional care group a proportion of children will receive appropriate treatment according to the probability of accessing high quality care. Children who access good quality care are assumed to be correctly diagnosed as having either malaria or non-malarial illness, and are assumed to receive appropriate treatment. Children without access to health care are assumed to go untreated and face a higher probability of developing severe illness. For children that develop severe illness in both arms, the mortality rates for the proportion of children who cannot access good quality care will be higher than in those with access to health services.

### Model design

**Figure 1:**
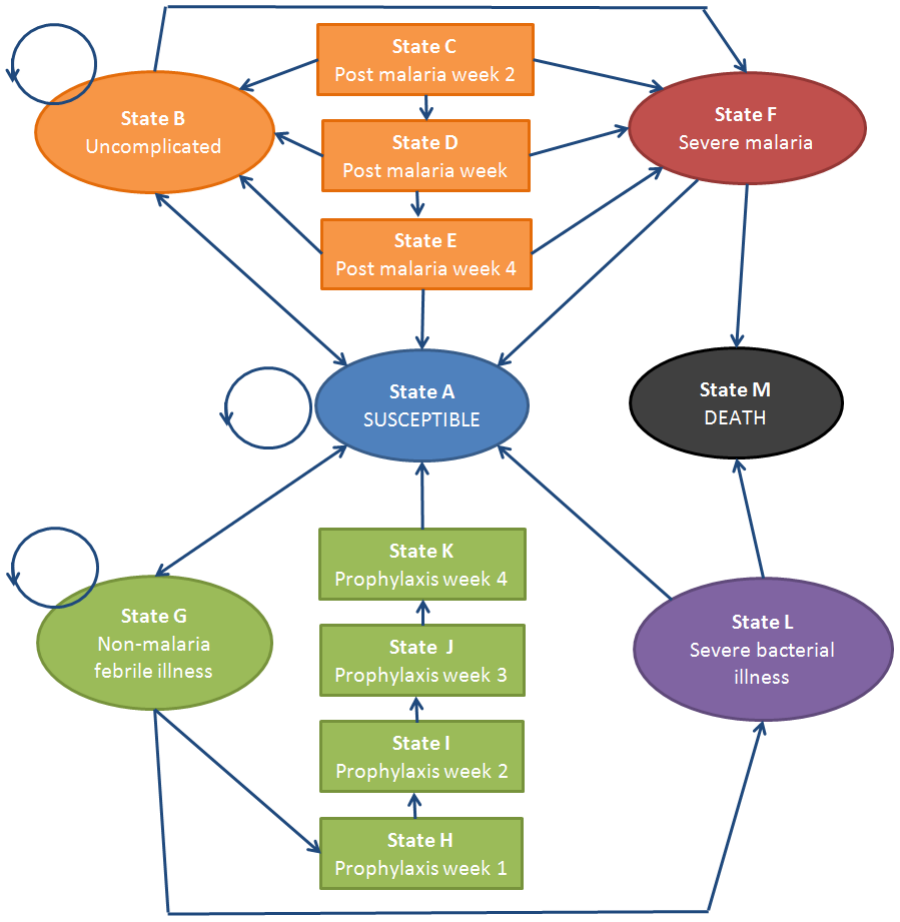
Illustration of the Markov model. Patients transition through the 13 states, each of which has its associated costs and health outcomes. The arrows depict which transitions can occur from one cycle to the next. The transition probabilities differ between patients that receive HMM and those who do not, and according to whether patients can access appropriate health care facilities.

The Markov model defines thirteen mutually exclusive health states representing childhood illness (Figure 1). Costs and health outcomes were attached to each health state, and transition probabilities were assigned to dictate the movement of children between the states over discrete time periods, or cycles [23]. The model was constructed using Microsoft Excel (2007) and macros were written with Microsoft Visual Basic® 6.3. The model was populated with data from Uganda, but is designed to be interactive, allowing the user to adjust certain parameters, including the drugs distributed in HMM and as first-line treatment for uncomplicated malaria in health facilities, and the perspective of the analysis. The transition probabilities and costs can also be adjusted.

### Markov states

The thirteen discrete health states included: State A: Susceptible, in which a child is healthy, but susceptible to illness; State B: Uncomplicated malaria; States C–E: Post-malaria weeks 2–4, representing the weeks following treatment for malaria during which a patient is at risk for recurrent malaria; State F: Severe malaria; State G: Non-malaria febrile illness; States H–K: Antimalarial prophylaxis weeks 1–4, representing the weeks following an antimalarial treatment during which a patient could benefit from a protective effect of the antimalarial; State L: Severe bacterial illness; and State M: Death. The arrows in the diagram represent the possible transitions that may occur between health states from one cycle to the next. The circular arrows alongside States A, B, and G indicate that a child may remain in the state s/he was in during the previous cycle.

### Transition probabilities

The probability of transitioning between each of the Markov states was estimated from data available in the literature, or where data were lacking, from expert opinion gathered in a Delphi survey (Table 1). The probability of developing uncomplicated malaria (State B) was determined from published estimates of the annual malaria incidence in children under five, ranging from 0.1 to 8 per episodes per child per year [15], [24]. The probability that a child with uncomplicated malaria (State B), who was appropriately treated, would develop severe malaria (State F), or die (State M), was determined from primary data collected in Kisiizi hospital in South West Uganda and from the literature [25], [26]. The probability that a child with uncomplicated malaria (State B), who was inappropriately, inadequately, or not treated, would develop severe malaria (State F), or die (State M), were determined from a Delphi survey [27].

**Table 1:** Parameter values.

Costs	Estimate				Source
**HMM distribution**	$0.2 per child/month				Primary data, Uganda MoH documents
**OPD care (excluding drugs)**	$4.5 per visit				Primary data – Jinja clinic
**Inpatient care for severe malaria**	$20 per stay (Based on average length of stay)				Primary data- Kisiizi Hospital
**Inpatient care for non-malaria severe illness**	$12 per stay (Based on average length of stay)				Primary data- Kisiizi Hospital
**Antimalarial costs**	HOMAPAK - $0.15 per febrile episode; AL - $0.65 per dose				Uganda MoH
**Antibiotic costs**	$0.3 per dose				[35]
**Transition probabilities**					
**Transmission**	**Low**		**High**		
**Untreated malaria becoming severe**	30% (10−58%)		13% (7−30%)		Delphi survey results for low and high transmission intensities [27]. In brackets are the inter-quartile ranges used in the sensitivity analyses. The medium transmission values in the model were an average of the high and low ones.
**CFR untreated severe malaria**	75% (50−85%)		60% (45−80%)		
**Proportion of NMFI that require antibiotics**	30% (10−40%)				
**Untreated bacterial NMFI becomes severe**	40% (18−73%)				
**CFR untreated severe NMFI**	50% (28−68%)				
**Disability adjusted life year (DALY) related parameters**					
**Life expectancy**	52 years				[40]
**Disability weights**	Non severe illness	Severe illness		Death	Global Burden of Disease disability weightings
	0.21	0.47		1	
**Discount rate**	3%				[41]

Assuming that a child with uncomplicated malaria was correctly treated, the probability that they would transition through the post-malaria states (States C–E) and become susceptible again (State A), or be at risk of another episode of uncomplicated malaria (State B) was based on the probability of malaria recurring due to recrudescence as determined by the efficacy of the antimalarial treatment, or re-infection, as determined by the level of malaria transmission and the duration of the post-treatment prophylactic effect of therapy. These probabilities were estimated using the risk of parasitemia, unadjusted by genotyping, as measured in antimalarial drug efficacy studies conducted in Uganda [28], [29], [30], [31], [32], [33], [34].

The probability that a child would develop a non-malarial febrile illness (State G) was based on estimates for the incidence of febrile episodes per year [25], subtracting the incidence of malarial episodes. The proportion of non-malaria febrile illnesses that would require antibiotic treatment (30%) was determined from the Delphi survey. The probabilities that a child with a non-malaria febrile illness (State G) who was appropriately treated would develop a severe bacterial illness (State L), or die (State M), were estimated from expert opinion as used in previous publications [35], [36]. The probabilities that a child with a non-malarial illness (State G) who did not receive appropriate antibiotic treatment would develop a severe bacterial illness (State L), or die (State M), were estimated from the Delphi survey.

The likelihood that a child with a non-malaria febrile illness would acquire a new malaria infection was based on malaria transmission intensity and the duration of the post-treatment prophylactic effect of therapy. Those children who received an antimalarial drug were assumed to be at lower risk due to the benefits of post-treatment prophylaxis (States H–K). The potential duration of post-treatment prophylaxis varied with the terminal elimination half-life and efficacy of the drugs, and was limited to four weeks in the model. Transition probabilities were estimated from the risk of new infection, adjusted by genotyping, as measured in antimalarial drug efficacy studies conducted in Uganda [28], [29], [30], [31], [32].

### Costing

The costs of Uganda's HMM programme were obtained from the Ministry of Health. The costs of purchasing and supplying antimalarial drugs, and training and monitoring community drug distributors (CDDs), were considered programme costs. In Uganda, CDDs are unpaid volunteers; the opportunity cost to their time was estimated from interviews with three CDDs. CDD time was assigned a monetary value equivalent to the Ugandan average wage [37].

The cost of providing good quality outpatient care was obtained from a clinic run by an international non-governmental organization in eastern Uganda. Costs were obtained for construction, overheads, and variable inputs, excluding the cost of providing AL, the current first line treatment for malaria in Uganda, which is included in the model separately. The costs of treating a child with severe illness were estimated from a mid-sized hospital run by a faith-based organization in south-western Uganda. Micro-costing, using patient records for treatments received and labour costs for staff, and step-down hospital costing were used to calculate departmental expenditure and to estimate the proportion of their services dedicated to pediatric care [38].

Household costs for management of febrile illness in children, including user fees, travel expenses, drugs purchased and other illness-related expenditures were obtained from the Kampala HMM trial [22]. Indirect costs including productivity losses due to the time caregivers spent away from their usual activities while caring for a sick child were costed using the average wage for unskilled workers [37]. Costs were collected in Ugandan Shillings and converted to US dollars (1USD  = 1686 Uganda Shillings for the year 2007). Costs were not discounted due to the relatively short time horizon.

The model permits analysis from either the provider or societal perspective. The provider perspective includes costs for HMM, and the costs of providing outpatient and inpatient care. The societal perspective includes provider costs, plus household costs and an additional cost for the potential harm of unnecessary antimalarial treatment. Such adverse consequences could include drug toxicity, spread of drug resistance, and use of scarce resources. The only available study to estimate this suggested that the provision of 200 antimalarial treatments will eventually result in the loss of one life in the future [39]. However, in our analysis a more conservative estimate of one loss of life per 600 antimalarial treatments was used as the potential harm of treatment, given that use of scarce resources is already accounted for in the drug costs. This value for the potential harm of treatment and most other parameters can be modified by the user to explore the impact on results.

### Antimalarial drugs

The model allows the user to select the drugs to be distributed in the HMM programme and those to be used as first-line treatment for uncomplicated malaria at the health facilities. Options for both include CQ+SP, amodiaquine + artesunate (AQ+AS), AL, and dihydroartemisinin-piperquine (DP). The model also permits the user to customize the characteristics of a regimen to be distributed through either pathway.

### Cost -effectiveness outcome

The model was designed to calculate the cost per disability adjusted life year (DALY) averted. Life expectancy in Uganda was estimated at 52 years, which was used to calculate the number of years of life lost for each death [40], discounted at 3% [41]. Disability weights were assigned according to the Global Burden of Disease weightings (0.21 for non-severe illness, 0.47 for severe illness, and 1 for death) [42], and were used to calculate the number of DALYs in each arm. A decision threshold equivalent to the 2007 Ugandan GDP per capita ($360) was used to determine when an intervention might be considered cost-effective [43], [44]. The model allows the user to adjust the decision threshold, including options of $25, $150, $360, and $720.

### Model output

A hypothetical cohort of 1000 children was run through the model using one-week cycles over five years. The model was constructed using the assumption that all individuals entered the model at time 0 in a healthy state. During each cycle, the transition probabilities were applied, and the distribution of patients in each of the health states was adjusted [23]. The costs and disability weights assigned to each health state are aggregated at the end of the analysis according to the total time children spend in each state.

**Figure 2:**
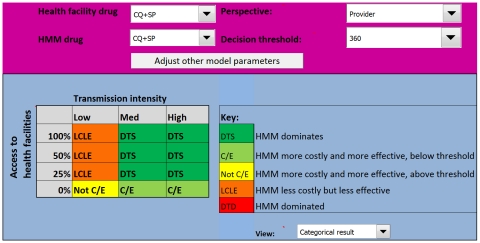
The model interface. The lower panel is the model output indicating the circumstances in which HMM is likely to be appropriate. Above this are the controls where the user can adjust the costs and transition probabilities, select drugs for both HMM and health facilities, determine the perspective for the analysis, and set the decision threshold.

The results of the analysis are presented across different levels of malaria transmission, stratified as low, medium, or high defined as an incidence of malaria episodes of 0.1 malaria cases a year in low transmission areas, 4 cases a year in medium transmission, and 8 cases in high transmission areas [45], [46], [47], [48], [49]. Results are also stratified by the probability that a child would receive appropriate medical care from a health facility, arbitrarily categorized as 0%, 25%, 50%, and 100% (Figure 2). This probability determined the proportion of children in the cohort that cycled through the model according to the transition probabilities for patients receiving appropriate treatment, while the remainder of the cohort is assumed to receive inadequate or no treatment, with their own transition probabilities (characterised by worse health outcomes).

The final outcome of the model is a product of the incremental cost-effectiveness ratio (ICER), and the ceiling ratio (the threshold value decision-makers are willing to pay to avert a DALY [43]), set at US$360 in this analysis [50]. Rather than presenting ICERs numerically, the results are categorized in manner to help guide policy-makers [37]:

- Outcome 1. HMM dominates, i.e. it is more effective and less costly (labelled *DTS*)
- Outcome 2. HMM is more effective but more costly, and the ICER is lower than the ceiling ratio suggesting the intervention is cost-effective (labelled *C/E*)
- Outcome 3. HMM is more effective, but more costly, however the ICER is higher than the ceiling ratio suggesting the intervention is not cost-effective (labelled *Not C/E*)
- Outcome 4. HMM is less costly but less effective (labelled *LCLE*)
- Outcome 5. HMM is dominated, i.e. it is less effective and more costly (Labelled *DTD*)

From a policy-maker's perspective, Outcomes 1 and 2 would unequivocally justify the adoption of the intervention, subject to budget availability. Outcome 3 suggests that although HMM is more effective, resources would be better used elsewhere. Outcome 4 indicates HMM is less effective, suggesting that the intervention should be rejected, even if it is less expensive, unless implementing such an intervention results in economic gains that outweigh the strong ethical objections to introducing a less effective intervention. Outcome 5 would suggest that HMM should be unequivocally rejected.

### Sensitivity analysis

The robustness of the results to variation in the input variables was assessed using sensitivity analyses. The impact of using different antimalarials in the HMM programme and at health facilities was also explored, including use of a hypothetical ‘ideal’ antimalarial, which was assumed to be inexpensive (cost equivalent to chloroquine), and 100% effective, providing post-treatment prophylaxis for 4 weeks. The model was initially run from the provider's perspective. In the sensitivity analysis, the societal perspective, including costs for carers, providers, and the harm of treatment factor, was used. Sensitivity analyses were also carried out for the values obtained in the Delphi survey using inter-quartile ranges for outcomes of untreated malaria, the proportion of non-malarial febrile illness that would benefit from antibiotic treatment, and the health outcomes for such illnesses if untreated [27].

## Results

### Costing

In Uganda's HMM programme, the drug costs constitute the majority of the overall programme costs, regardless of the regimen (Table 2). Replacing CQ+SP with AL increases the drug costs and total cost per child considerably. The average cost for providing high quality outpatient care was $4.5 per patient visit. Inpatient care costs of treating severe illnesses were estimated to be $11.2 per hospital stay for malaria and $20.4 per stay for non-malaria illnesses. Treatment for malaria tended to be less expensive due to shorter duration of hospitalization and lower costs of antimalarial drugs relative to the treatments provided other patients. The household costs of treating febrile episodes were lower with HMM programme, compared to conventional care (no HMM), averaging $0.90 and $1.50, respectively. The amount of time caregivers spent away from their usual activities while their child was ill was slightly lower with an HMM programme (0.85 days) than without (0.96 days). Including these indirect opportunity costs, the total household costs per episode of childhood fever were $1.90 with HMM and $2.80 without.

**Table 2:** Costing results.

	CQ+SP		AL	
	Monthly cost per child	Annual cost per child	Monthly cost per child	Annual cost per child
Staff (CDDs)	$0.05	$0.60 (24%)	$0.05	$0.60 (7%)
Provider cost (MoH)	$0.05	$0.12 (5%)	$0.05	$0.12 (1%)
Drugs	$0.01	$1.80 (71%)	$0.65	$7.80 (92%)
**TOTAL**	**$0.21**	**$2.52**	**$0.71**	**$8.52**

### HMM distributing CQ+SP

**Figure 3:**
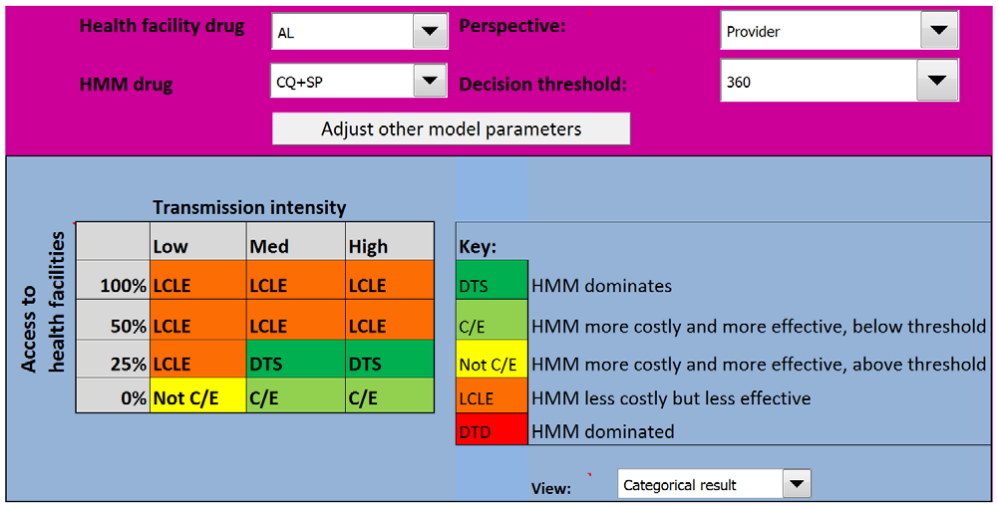
Model output for the Uganda HMM programme using CQ+SP from a provider perspective. The model suggests that HMM will only be efficient in areas of medium and high transmission, where the probability of appropriate care is low. In low transmission areas HMM is more effective but too costly, and is not cost-effective. CQ+SP  =  chloroquine + sulfadoxine-pyrimethamine.

In the baseline analysis, CQ+SP was selected for HMM, and AL was selected for first-line treatment for uncomplicated malaria at the health facilities. The model output chart (Figure 3) indicates that the cost-effectiveness of HMM using CQ+SP from the provider perspective varies with malaria transmission and the probability that a child would receive appropriate care. HMM is most attractive in medium to high malaria transmission settings, where the probability of appropriate care is limited, as evidenced by the placement of Outcomes 1 and 2 on the figure. In low transmission areas, HMM is only more effective than the alternative if the probability of appropriate care is zero; however, here the HMM intervention is more costly, and the ICER is higher than the ceiling ratio therefore it is not deemed cost-effective (Outcome 3). HMM is less costly but less effective (Outcome 4) than the alternative in low transmission settings if the probability of appropriate care is 25% or greater, and in medium to high transmission if that probability is 50% or above.

### HMM distributing AL

**Figure 4:**
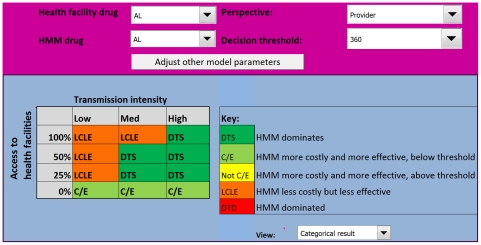
Model output for the Uganda HMM programme using AL from a provider perspective. Introducing AL into HMM appears to be efficient in most medium to high transmission areas, unless the probability that a child will receive appropriate care from a health facility is 100%. AL  =  artemether-lumefantrine.

Replacing CQ+SP with AL in HMM changes the output of the model considerably (Figure 4). With AL, HMM becomes attractive from the provider perspective in several areas where it was previously less effective than the alternative. In high transmission areas, HMM is more effective and less costly (Outcome 1) in all settings, except where the probability of appropriate care is zero; here HMM is more costly, but remains more effective (Outcome 2). In medium transmission settings, HMM with AL is more effective (Outcomes 1 and 2) if the probability of appropriate care is 50% or less, only becoming less effective (Outcome 4) when the probability of access to appropriate care is 100%. With AL, HMM remains more costly, but becomes more effective (Outcome 2) in low transmission settings where there is no chance of appropriate care. Even with AL, HMM is less effective than the alternative in low transmission settings if the probability of appropriate care is 25% or greater.

### Sensitivity analysis

**Figure 5:**
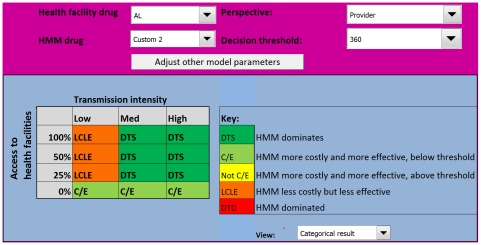
Model output for the Uganda HMM programme using a hypothetical ‘ideal’ antimalarial from a provider perspective. These results indicate that even if an ‘ideal’ antimalarial is introduced into HMM, the settings where HMM is cost-effective remain limited to medium and high transmission areas, unless the probability of receiving appropriate care is zero.

**Figure 6:**
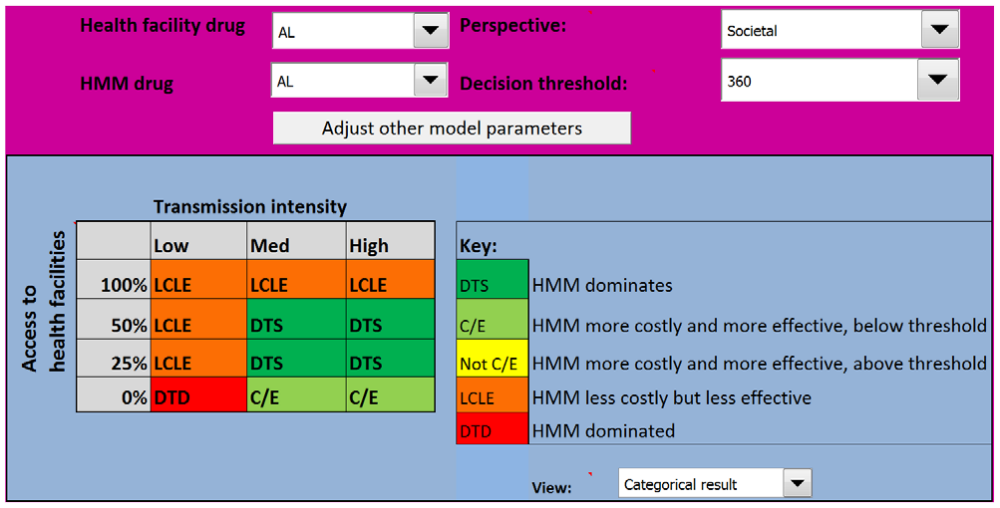
Model output for the Uganda HMM programme using AL from a societal perspective. Under this scenario HMM is only warranted in medium to high transmission areas and where the probability of appropriate care is low.

**Figure 7:**
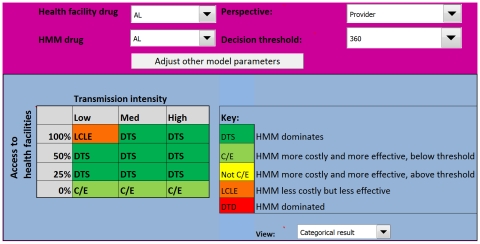
Model output for the Uganda HMM programme using AL in the context of a lower burden of non-malaria febrile illnesses. When the proportion of non-malaria febrile illnesses are assumed to require less antibiotics and their health outcomes is estimates as less severe HMM appears more beneficial in almost all areas.

When a hypothetical ‘ideal’ antimalarial regimen was introduced into HMM, with AL as the first-line treatment distributed at health facilities, the output of the model changed little from the provider perspective (Figure 5). Only in the medium transmission setting where the probability of appropriate care was 100% did the HMM intervention change from less effective (Outcome 4) to more effective and highly attractive (Outcome 1). Otherwise, the ICER output categories remained similar to those for HMM with AL (Figure 4). When the model was run from the societal perspective, including costs to the household and the potential harm of unnecessary antimalarial treatment, HMM with AL became unattractive in several areas (Figure 6). Where the probability of appropriate care was over 50%, HMM became less effective (Outcome 4) from a societal perspective, regardless of transmission intensity. In low transmission settings where the probability of appropriate care was zero, HMM went from more effective (Outcome 2) to not effective (Outcome 5). Using estimates for better and worse health outcomes for untreated malaria according to the inter-quartile ranges in the Delphi survey had no effect on the model outcome. Reducing the proportion of NMFIs that require antibiotics and the probability that these result in death when untreated, improved the cost-effectiveness of HMM greatly, as shown in Figure 7. Using higher estimates for the proportion of NMFIs that require antibiotics and the probability of death for untreated NMFIs did not have a significant effect.

## Discussion

A Markov model was constructed as a decision support tool to estimate the cost-effectiveness of HMM as compared to conventional care for febrile illnesses in children in Uganda. The analysis indicates that the cost-effectiveness of HMM varies with malaria transmission and the probability that a child will receive appropriate care from a health facility. Where transmission is high and the probability of appropriate care is limited, HMM is likely to be cost-effective from a provider perspective. Replacing CQ+SP with AL in HMM expands the range of beneficial coverage, but HMM only remains a highly attractive intervention in areas of high malaria transmission, and in medium transmission areas with a lower probability of appropriate care. Considering the analysis from the societal perspective decreases the attractiveness of HMM with AL. HMM is generally not cost-effective in low transmission areas, regardless of which antimalarial is distributed. HMM may be a useful strategy for higher transmission settings with limited health care infrastructure, allowing carers to obtain effective antimalarials without having to transport their children to far away facilities, but is not appropriate for all settings.

HMM programmes have been criticized for focusing only on treatment of malaria [17], [51], [52]. Expanding programmes to provide more comprehensive care, including treatment for respiratory illnesses and diarrhea has been suggested. In Uganda, there are plans to integrate HMM into a programme of integrated community case management (iCCM), in which village health teams (VHTs) will provide presumptive treatment for malaria, pneumonia, and diarrhea based on clinical criteria. iCCM addresses the issue that not all fevers are due to malaria, and is promoted by WHO [53], [54]. However, this broader programme still relies on presumptive treatment, and evidence supporting the health impact, feasibility, and sustainability of iCCM are lacking. The introduction of rapid diagnostic tests (RDTs) for malaria into these programmes could enhance their effectiveness and improve the targeting of both antimalarials and antibiotics.

There is increasing evidence that malaria transmission and the burden of disease is decreasing in many areas of Africa [55], [56], [57], [58], [59]. Although the reasons for this change are multi-factorial and not always clear, there is optimism that the trend will continue in at least a significant part of malaria-affected Africa. Where malaria transmission is reduced, and the proportion of febrile illnesses that are attributable to other illnesses increases, this study demonstrates the strategy of presumptive treatment of all fever cases for malaria becomes much less attractive. Indeed, the World Health Organization has recently released new guidelines regarding treatment of malaria, now recommending that suspected cases of malaria be confirmed by a parasitological test, when possible [60]. The move towards universal diagnostic testing is a major shift in malaria case management. Introduction of RDTs for malaria into lower level health facilities and at the community-level is currently being explored to expand diagnostic capability; however, whether RDTs can be successfully utilized in the periphery remains unclear [61].

The Markov model used in this analysis has several limitations. Although the health states and transitions were designed to mimic real scenarios, the model remains artificial and has to make simplifications, as do all models. Transitions between health states were restricted to limit the complexity of the model. The model did not account for the acquisition of antimalarial immunity in children under five, which may occur early in high transmission areas [62]; this would if anything reduce the effectiveness of HMM. The model assumed that all children would be at equal risk of acquiring malaria, regardless of age, which is an oversimplification in high-transmission settings, but is difficult to quantify. Immunity in older children would imply that HMM is a less effective strategy for these children, as many of those with severe symptoms are likely to have other, non-malarial causes of illness. Neurological sequelae following episodes of severe malaria were not accounted for, as including this consequence in the model complicated it considerably and had negligible impact on outcomes. Deaths from unrelated causes were also excluded as these were assumed to be constant with or without HMM. The probability that a child would receive appropriate medical care from a health facility was also oversimplified in the model and the stratification was arbitrary. Similarly, a different stratification of the levels of access to care and the transmission intensity would result in slightly different outcomes, however the overall message, that HMM becomes a less cost-effective strategy as access to care improves and transmission decreases, remains the same. A more precise assessment of the cost-effectiveness of HMM in different settings would require additional information about the different steps of the pathway to appropriate care, including availability of health services and malaria diagnostics, utilization, and quality of care. The potential harm of unnecessary antimalarial treatment including the risks of drug toxicity, spread of drug resistance, and misuse of scarce resources, included in the societal costs, is very challenging to value. Although consideration of these adverse consequences is essential when weighing the benefits and risks of HMM, significant uncertainty exists around this parameter, and further research is needed to provide more accurate estimates. These potential weaknesses are however unlikely to affect the key outcomes of the model.

### Conclusions

HMM programmes are being implemented across Africa, but evidence of their cost-effectiveness is limited. This analysis suggests that the cost-effectiveness of HMM varies substantially with malaria transmission intensity and the probability that a child will receive appropriate care at a health facility. Results of the model suggest that adopting a policy of HMM with AL is justified from the provider perspective in high transmission areas, regardless of access to care; in most medium transmission areas, unless the probability of appropriate care is 100%; and in low transmission areas only if there is no chance of appropriate care. The analysis suggests that HMM should not be adopted for use in other settings, where the programme might be less effective and/or more costly. Comprehensive implementation of HMM could result in poor health outcomes and misallocate valuable resources. HMM and other community-based strategies may need to be tailored to specific settings, accounting for local malaria transmission intensity and availability of health services.

## Supporting Information

Model S1 Interactive model for the evaluation of HMM strategies. The model is a Microsoft Excel file and requires that macros are allowed to run.
(0.57 MB XLS)
